# mRNA expression of CK19, EGFR and LUNX in patients with lung cancer micrometastasis

**DOI:** 10.3892/etm.2013.1417

**Published:** 2013-11-19

**Authors:** XIANSEN ZHANG, JING XIE, CHANGFA YU, LIXIA YAN, ZESHAN YANG

**Affiliations:** Department of Clinical Laboratory, Taizhou First People’s Hospital, Taizhou, Zhejiang 318020, P.R. China

**Keywords:** cytokeratin 19, epidermal growth factor receptor, lung-specific X protein, lung cancer, micrometastasis

## Abstract

To evaluate the clinical significance of mRNA expression of cytokeratin 19 (CK19), epidermal growth factor receptor (EGFR) and lung-specific X protein (LUNX), a total of 42 patients who were diagnosed with non-small cell lung cancer (NSCLC) by pathology were studied retrospectively. The messenger RNA (mRNA) expression levels of CK19, EGFR and LUNX in the peripheral blood were analyzed using reverse transcription-polymerase chain reaction (RT-PCR). The expression of CK19 mRNA did not differ significantly according to the location, size, clinical stage or differentiation of the primary tumor (all P>0.05). However, there was a significant difference in the level of CK19 mRNA expression between squamous carcinoma and adenocarcinoma. The positive rates of EGFR mRNA in the patient and the healthy control groups were 69.0 and 12.5%, respectively, and were significantly different (P<0.05). The positive rates of LUNX mRNA in the two groups were 40.5 and 0%, respectively, and were significantly different (P<0.05). The results indicate that the mRNA expression of CK19, EGFR and LUNX in the peripheral blood is of significant clinical value for the diagnosis of micrometastasis and the prognosis of lung cancer.

## Introduction

Lung cancer is one of the most malignant types of tumor and presents a serious threat to the health of human beings. In recent years, significant progress has been made in the treatment of lung cancer; however, the therapies remain unsatisfactory. The overall five-year survival rate for non-small cell lung cancer (NSCLC; all stages combined) is ~15%, and even for patients in the early stage of the disease, who have undergone radical excision, the survival rate is only 50–70% (1). The reasons behind the failure of therapy are recurrence and metastasis following surgery, which may be associated with metastasis via the blood or the lymphatic system in the early stage. Micrometastasis is an indication of a high possibility of recurrence and metastasis, and is associated with the pathological pattern, grade of differentiation and tumor node metastasis (TNM) stage. The early discovery of micrometastasis has an important clinical significance in recurrence and the prognostic evaluation of lung cancer. In addition, it has particular value for improving the prognosis of patients (2). At present, reverse transcription-polymerase chain reaction (RT-PCR) is used for the detection of micrometastases from solid tumors and to quantify the expression of tumor markers and genes associated with tumors in the peripheral blood. In the present study, the messenger RNA (mRNA) expression levels of cytokeratin 19 (CK19), epidermal growth factor receptor (EGFR) and lung-specific X protein (LUNX) were assessed and the clinical significance of the mRNA levels was evaluated.

## Materials and methods

### Patients

A total of 42 patients (27 males and 15 females; age, 23–81 years; average age, 58.1 years), who were diagnosed with NSCLC by pathology from May 2008 to April 2011, were studied retrospectively in Taizhou First People’s Hospital (Taizhou, China). None of the patients presented with second primary tumors and all the patients received initial treatment. There were 25 patients with squamous carcinomas and 17 patients with adenocarcinomas. According to the TNM criteria, revised by the International Union Against Cancer (UICC) in 1997, there were 4 cases in stage I, 12 cases in stage II, 17 cases in stage III and 9 cases in stage IV. Furthermore, there were 19 patients with well-differentiated, 14 patients with moderately differentiated and 9 patients with poorly differentiated lung cancer. The positive control group included four patients undergoing surgery for NSCLC. The healthy control group included 40 individuals (27 males and 13 females; age, 25–79 years; average age, 52.35 years), while the control group included 40 patients (23 males and 17 females; age, 27–75 years; average age, 55.6 years) with a benign disease. Of these 40 control patients, 15 patients had pneumonia, 8 patients had bronchiectasia, 4 patients had a lung abscess, 3 patients had a tuberculoma and 10 patients had bullae of the lung. This study was approved by the Ethics Committee of Taizhou First People’s Hospital (Taizhou, China) and all participants gave written informed consent.

### Reagents and experimental apparatus

Lymphocyte separation medium was purchased from Shanghai Hengxin Chemical Reagent Co, Ltd. (Shanghai, China) and TRIzol™ reagent was obtained from Gibco (Carlsbad, CA, USA). Taq DNA polymerase and a First Strand cDNA Synthesis kit were purchased from Toyobo (Osaka, Japan). DNA marker was obtained from Beijing Dingguo Changsheng Biotechnology Co., Ltd. (Beijing, China). The LightCycler 480 instrument used for the RT-PCR assays was from Roche (Mannheim, Germany). The Multiskan Spectrum was purchased from Thermo Fisher (Waltham, MA, USA). The UV transmission detection analyzer (ZF-3) was purchased from Shanghai Jihui (Shanghai, China)

### Blood samples

Prior to any treatment, a tube containing 5 ml fasting peripheral venous blood was collected from each patient. Following this, ethylenediaminetetraacetic acid (EDTA) was added for anticoagulation. The mRNA expression levels of CK19, EGFR and LUNX in the blood samples were subsequently assessed.

### Extraction of total RNAs

Karyocytes were isolated from the blood samples using lymphocyte separation medium and transferred into an Eppendorf tube. Following centrifugation, the supernatant was discarded, 1,500 μl TRIzol reagent was added and the sample was mixed thoroughly and kept at room temperature for 10 min. Chloroform (100 μl) was then added and the sample was centrifuged for 10 min at 6,708 × g and 4°C. The supernatant was transferred to a 1.5-ml EP tube and, following the addition of isopropanol, was maintained at 0°C for 20 min. The volume of isopropanol was 3-fold that of the supernatant. The supernatant and isopropanol were subsequently centrifuged at 15,093 × g for 15 min, and then the supernatant was discarded. The RNA precipitate was washed once with 200 μl 75% ethanol, centrifuged, dehumidified and dissolved by adding 20 μl diethylpyrocarbonate (DEPC). Following surgery, the NSCLC tissues were cryopreserved in liquid nitrogen. A total of 50 mg NSCLC tissues was used for grinding and the total RNAs were extracted by the method described above. All the samples were preserved at −70°C. The concentration and purity were measured using the Multiskan Spectrum spectrophotometer. A260/A280 ratios in the range of 1.8 to 2.0 were considered satisfactory for purity standards in this study.

### Synthesis of cDNA

The reverse transcription system (10 μl) included 2.0 μl 5X PrimeScript™ Buffer, 0.5 μl random 6 mers (100 μmol/l), 0.5 μl PrimeScript™ RT enzyme Mix I, 0.5 μl oligo(dT) primer (50 μmol/l), 500 ng total RNA and RNase-free dH_2_O. The reaction conditions were 37°C for 15 min, followed by 85°C for 5 sec. cDNA samples were preserved at −20°C.

### RT-PCR

The primers were as follows: for GAPDH: (sense: 5′-GGCTGGGACTGGCTGAGCCT-3′ and antisense: 5′-TGGCGACGCAAAAGAAGATG-3′); for CK19: (sense: 5′-GAAATCAGTACGCTGAGGGG-3′ and antisense: 5′-CCGGCTGGTGAACCAGGCTT-3′); for EGFR: (sense: 5′-AAATCCTGCATGGCGCCGTG-3′ and antisense: 5′-GGTGGTTCTGGAAGTCCATC-3′); for LUNX: (sense: 5′-AATGAGGTTCTCAGAGGCTT-3′ and antisense: 5′-TTAGACCTTGATGACAAACT-3′). The quantitative PCR system (20 μl) included 10.0 μl 2X SYBR Premix Ex Taq, 0.4 μl forward primer (10 μmol/l), 0.5 μl reverse primer (10 μmol/l), 2.0 μl cDNA and 7.2 μl ddH_2_O. Glyceraldehyde 3-phosphate dehydrogenase (GAPDH) was used as an internal control. The PCR conditions were as follows: 40 cycles of predenaturation at 95°C for 1 min, denaturation at 95°C for 5 sec and annealing and extension at 62°C for 5 sec. Every sample required two duplicated tubes, and two tubes of positive control and one tube of negative control (without cDNA template) were included.

### Gel electrophoresis

Following amplification, agarose gel electrophoresis and ethidium bromide (EB) staining were performed. An ultraviolet (UV) transmission instrument was used to record the results.

### Standard curve

The cDNA samples of the NSCLC tissues were diluted to the following gradients: 10^0^, 10^1^, 10^2^ and 10^3^. Following RT-PCR, amplification curves were generated, respectively. The cDNA of all the samples in the 20-μl PCR reaction solutions amounted to the cDNA obtained from reverse transcription with different total RNA (100, 10, 1 and 0.1 ng respectively). The initial copy numbers of the mRNA in the samples were set as 100, 10, 1 and 0.1, respectively, prior to the standard curve being constructed by the LightCycle 480 instrument, of formula: Y = KX + B, where X is the logarithm of the initial copy number and Y is the value of the cycle threshold.

### Repetitive experiments

Three tubes of positive control and one tube of negative control were analyzed eight times, respectively, and the value of the cycle threshold (Ct) was determined. The mRNA expression levels of GAPDH, CK19, EGFR and LUNX mRNA were determined.

### Relative quantitative analysis

The positive control had an initial copy number of 100, and the relative expression of the target gene in a sample (F) was defined as the ratio of the expression of the target gene in the sample to that of the positive control. The expression of the target gene in a sample was standardized using GAPDH as an internal reference. From the standard curve, the initial copy number of the target gene in a sample was obtained. The relative expression of the target gene of a sample was then calculated using the formula: 10ΔYt/Bt - ΔYg/Bg (where ΔYt is the difference in the target gene Ct value between the sample and the positive control, ΔYg is the difference in the GAPDH Ct values between the sample and positive control, Bt is the slope of the standard curve of the target gene of the sample and Bg is the slope of the standard curve of GAPDH).

### Statistical analysis

SPSS 15.0 statistical software (SPSS, Inc., Chicago, IL, USA) was used for data analysis. The quantitative data were analyzed using one-way analysis of variance (ANOVA) and χ^2^ tests. P<0.05 was considered to indicate a statistically significant difference.

## Results

### RT-PCR and gel electrophoresis

**Figure 1:**
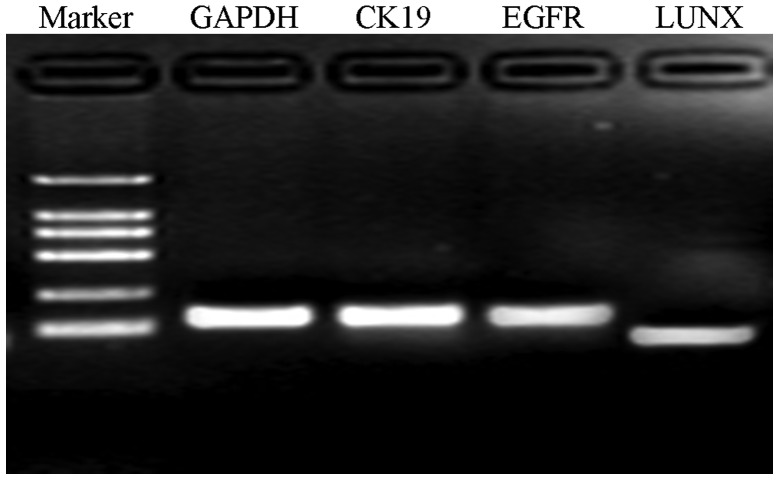
Gel electrophoresis of glyceraldehyde 3-phosphate dehydrogenase (GAPDH), cytokeratin 19 (CK19), epidermal growth factor receptor (EGFR) and lung-specific X protein (LUNX) messenger TNA (mRNA).

As shown in Fig. 1, in the evaluation of the distribution of the mRNA, it was indicated that the lengths of GAPDH, CK19, EGFR and LUNX mRNA were 150, 130, 126 and 90 bp, respectively. No evident dimer was observed (Fig. 1).

### Standard curve

With regard to the amplification curves of the positive samples, samples with different initial concentrations had different Ct values, which was the beginning of the exponential stage of PCR and the gradient (from left to right the initial copy numbers were 100, 10, 1 and 0.1, respectively). If the difference was approximated and the initial concentration was considerably higher, the Ct value was likely to be much smaller. With regard to the standard curve, there was a linear correlation between the Ct value (Y) and the logarithm of the copy number of the sample (X). The correlation coefficient was always 1. It was possible to obtain the initial copy number of the sample from the standard curve from the Ct value.

**Figure 2:**
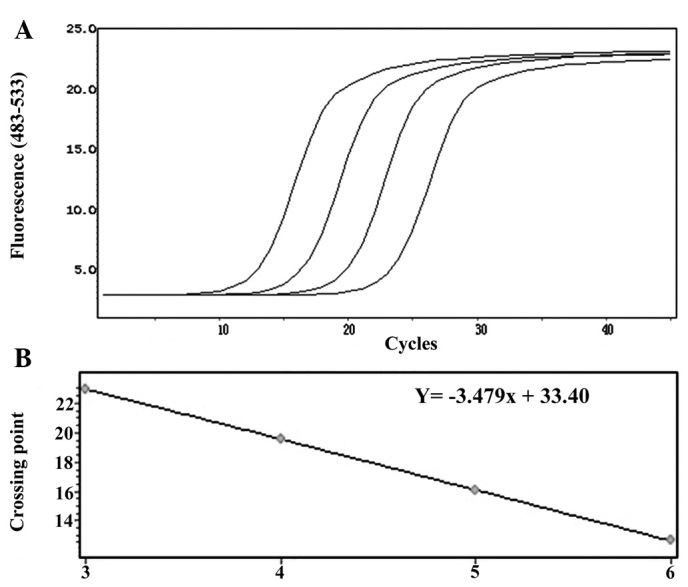
The (A) amplification and (B) standard curves of glyceraldehyde 3-phosphate dehydrogenase (GAPDH).

**Figure 3:**
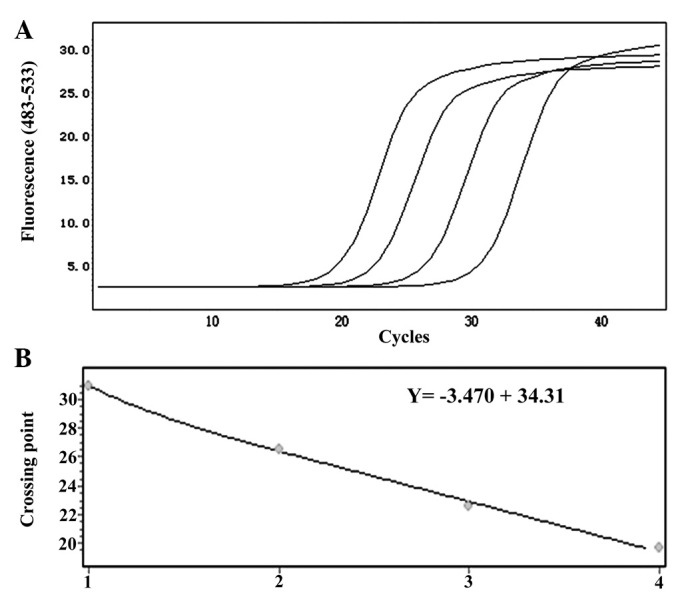
The (A) amplification and (B) standard curves of cytokeratin 19 (CK19) messenger RNA (mRNA).

**Figure 4:**
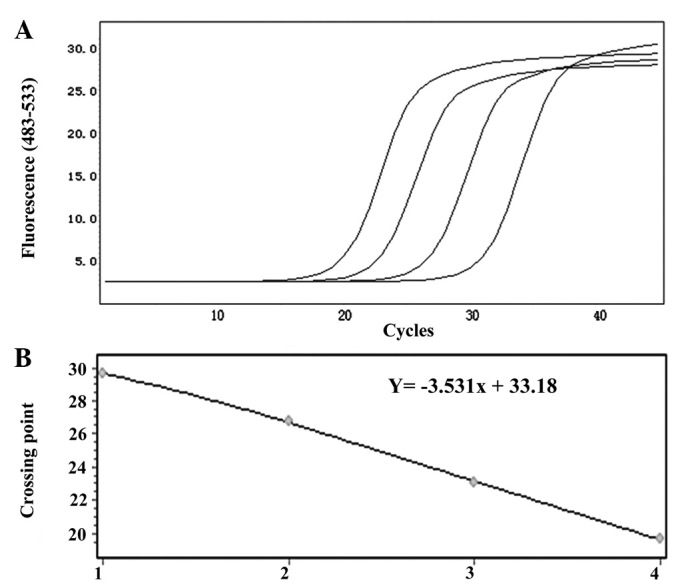
The (A) amplification and (B) standard curves of epidermal growth factor receptor (EGFR) messenger RNA (mRNA).

**Figure 5:**
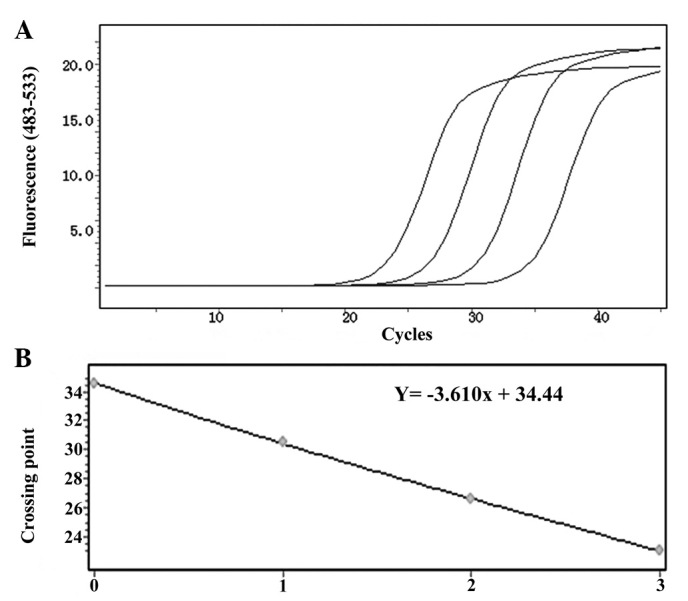
The (A) amplification and (B) standard curves of lung-specific X protein (LUNX) messenger RNA (mRNA).

With regard to the amplification curve of GAPDH (Fig. 2A), the standard curve had good correlation (Fig. 2B) and the regression equation was Y = −3.479× + 33.40. Furthermore, as shown in Fig. 3A, the amplification curve of CK19 mRNA and the standard curve showed good correlation (Fig. 3B). The amplification and standard curves of EGFR and LUNX mRNA are shown in Figs. 4 and 5, respectively.

The results of the repeated experiments are shown in Table I. These results indicated that the Ct values of GAPDH, CK19 mRNA, EGFR mRNA and LUNX mRNA had high repeatability and stability. The result of the healthy control group was negative.

### Relative quantitative analysis

The positive rates of CK19 mRNA in the patient and healthy control groups were 76.2 (32/42) and 15.0% (6/40) respectively, which showed a significant difference (P<0.05). The relative expression levels of CK19 mRNA were 1.72±0.41 and 0.27±0.13, respectively, in the two groups, which showed a significant difference (P<0.05). The expression level of CK19 did not differ significantly according to the location, size, clinical stage, differentiation of the primary tumor (all P>0.05). The relative expression level of CK19 mRNA was higher for central lung cancer, T3 + T4, stages III and IV and poorly differentiated lung cancer, although with no significant differences (all P>0.05). A significant difference was observed between the expression of CK19 mRNA in squamous carcinoma and adenocarcinoma (P<0.05), with a higher expression level in squamous carcinoma. The positive rates of EGFR mRNA in the patient and healthy control groups were 69.0 (29/42) and 12.5% (5/40) respectively, which showed a significant difference (P<0.05). The positive rates of LUNX mRNA in the patient and healthy control groups were 40.5 (17/42) and 0% (0/40) respectively, which showed a significant difference (P<0.05; data not shown). Expressions of LUNX mRNA were observed in 3 cases of stage I and II NSCLC, and 6 cases of stage IV NSCLC.

## Discussion

CK is a key component of intermediate fibers of the cytoskeleton of epithelial cells and is expressed in normal epithelial cells, epithelial tumors and metastatic cells (3). A false positive for CK expression may be observed due to the contamination of epithelial cells, the interference of a pseudogene and the low expression level of CK19 mRNA in the peripheral blood (4). In order to avoid the contamination of epithelial cells caused by blood collection in the present study, vacuum blood collection was used in the clinic and a second tube of blood was used to analyze the expression of CK19 mRNA. In the present study, the results indicated that the expression of CK19 mRNA was associated with the pathology of NSCLC. There was a significant difference in the level of CK19 mRNA expression between squamous carcinoma and adenocarcinoma. As a type of molecular marker for the micrometastasis of NSCLC in the peripheral blood, CK19 mRNA was shown to be important for the diagnosis of micrometastasis. However, studies with large samples and follow-ups are required to improve the specificity.

EGFR is the coreceptor of EGF and transforming growth factor (TGF). The epidermal growth factor receptor is commonly overexpressed in NSCLC. Since there are only small numbers of tumor cells in micrometastases, it has not previously been possible to use the routine examination of cell morphology to detect tumor cells due to a low sensitivity (2). However, with the development of molecular biology and immunology, tumor cells of micrometastases have been able to be detected. The technologies of immunohistochemistry, flow cytometry and RT-PCR have been applied for the detection of the molecular markers of micrometastasis or tumor cells in the lymph nodes, marrow and peripheral blood. The positive rate of molecular markers has clinical value as a predictive indicator of the prognosis (5).

LUNX is a novel human lung-specific gene that was isolated by differential-display mRNA analysis in a study by Iwao *et al*(6). The results of that study indicated that NSCLC tumors and cancer-free lung tissues were positive for LUNX mRNA. LUNX mRNA expression was enhanced in NSCLC tumors. There was no expression of LUNX mRNA in the cells of the peripheral blood. If LUNX mRNA was detected in the peripheral blood, it indicated that there were tumor cells and micrometastasis in the circulation (6). The study by Cheng *et al*(7) provided a detailed evaluation of the lung cancer tumor markers of LUNX, CK19, carcinoembryonic antigen (CEA), vascular endothelial growth factor (VEGF-C) and heterogeneous nuclear ribonucleoprotein (hnRNP) A2/B1 mRNA and assessed the diagnostic utility of these markers in patients with NSCLC. The results indicated that LUNX mRNA was the most specific gene marker for lung cancer and had potential diagnostic utility when measured in the peripheral blood and pleural fluid of patients with NSCLC (7).

In the present study the results showed that the positive rate of LUNX mRNA in NSCLC patients was 40.5% (17/42). There were 3 cases showing the expression of LUNX mRNA out of 16 cases of stage I and II NSCLC, which indicated that there were micrometastases in the peripheral blood in the early stages of NSCLC. For the control group (with benign disease), there was no expression of LUNX mRNA, which showed that the detection of LUNX mRNA had high specificity. In the present study, there were 6 cases that tested positive for LUNX mRNA expression out of 9 cases of stage IV NSCLC, which indicated that the possibility of micrometastasis in the peripheral blood increased in the advanced stage. There have been a number of studies concerning the diagnosis of lung cancer micrometastasis using the detection of LUNX mRNA (6,8). The results of the study by Iwao *et al*(6) showed that LUNX mRNA was detected in 16/20 (80%) histologically positive lymph nodes and 21/84 (25%) histologically negative lymph nodes (6). The results of the study by Yang *et al*(8) revealed that LUNX mRNA was detected in the peripheral blood and regional lymph nodes, and that there was no expression of LUNX mRNA in benign lung diseases and the peripheral blood and lymph nodes of healthy people. Therefore, the evaluation of mRNA expression of CK19, EGFR and LUNX in the peripheral blood had important clinical value for the diagnosis of micrometastasis and the prognosis of lung cancer.

## Figures and Tables

**Table I:** Results of repetitive experiments.

Analyte	Mean of Ct value	Standard deviation	Coefficient of variation
GAPDH	22.19	0.249	0.011
CK19	27.11	0.162	0.006
EGFR	25.61	0.216	0.008
LUNX	28.22	0.045	0.002

CK19, cytokeratin 19; EGFR, epidermal growth factor receptor; LUNX, lung-specific X protein.
